# Clr-f expression regulates kidney immune and metabolic homeostasis

**DOI:** 10.1038/s41598-022-08547-9

**Published:** 2022-03-22

**Authors:** Haggag S. Zein, Elias Abou-Samra, Michal Scur, Alex Gutsol, Clayton W. Hall, Bishal Dasgupta, Lara Gharibeh, Turki Abujamel, Daniel Medina-Luna, Gayani S. Gamage, Tessa J. Pelino, Mona Nemer, Mir Munir A. Rahim, Alexander Steinle, Brendon D. Parsons, Andrew P. Makrigiannis

**Affiliations:** 1 Department of Microbiology and Immunology, Dalhousie University, Halifax, NS Canada; 2 King Abdullah International Medical Research Centre, Immunology Research Program, Riyadh, Saudi Arabia; 3 Department of Biochemistry, Microbiology, and Immunology, University of Ottawa, Ottawa, ON Canada; 4 Division of Nephrology, Department of Medicine, Kidney Research Centre, Ottawa Hospital Research Institute, University of Ottawa, Ottawa, ON Canada; 5 Department of Pathology and Molecular Medicine, Division of Medical Microbiology, McMaster University, Hamilton, ON Canada; 6 Molecular Genetics and Cardiac Regeneration Laboratory, Department of Biochemistry, Microbiology, and Immunology, University of Ottawa, Ottawa, ON Canada; 7 Division of Cardiac Surgery, University of Ottawa Heart Institute, Ottawa, ON Canada; 8 Vaccines and Immunotherapy Unit, King Fahd Medical Research Center, King Abdulaziz University, Jeddah, Saudi Arabia; 9 Department of Medical Laboratory Technology, Faculty of Applied Medical Sciences, King Abdulaziz University, Jeddah, Saudi Arabia; 10 Beatrice Hunter Cancer Research Institute, Halifax, NS Canada; 11 Department of Biomedical Sciences, University of Windsor, Windsor, ON Canada; 12 Institute for Molecular Medicine, Goethe University Frankfurt, Frankfurt am Main, Germany; 13 Frankfurt Cancer Institute, Goethe University Frankfurt, Frankfurt am Main, Germany; 14 Department of Microbiology and Immunology, Dalhousie University, Sir Charles Tupper Medical Building, Rm. 7-F2, 5850 College Street, Halifax, NS B3H 4R2 Canada; 15 Department of Microbiology and Immunology, Dalhousie University, Sir Charles Tupper Medical Building, Rm. 7-B2, 5850 College Street, Halifax, NS B3H 4R2 Canada

**Keywords:** Chronic kidney disease, Innate immunity

## Abstract

The C-type lectin-related protein, Clr-f, encoded by *Clec2h* in the mouse NK gene complex (NKC), is a member of a family of immune regulatory lectins that guide immune responses at distinct tissues of the body. Clr-f is highly expressed in the kidney; however, its activity in this organ is unknown. To assess the requirement for Clr-f in kidney health and function, we generated a Clr-f-deficient mouse (*Clr-f*^−/−^) by targeted deletions in the *Clec2h* gene. Mice lacking Clr-f exhibited glomerular and tubular lesions, immunoglobulin and C3 complement protein renal deposits, and significant abdominal and ectopic lipid accumulation. Whole kidney transcriptional profile analysis of *Clr-f*^−/−^ mice at 7, 13, and 24 weeks of age revealed a dynamic dysregulation in lipid metabolic processes, stress responses, and inflammatory mediators. Examination of the immune contribution to the pathologies of *Clr-f*^−/−^ mouse kidneys identified elevated IL-12 and IFNγ in cells of the tubulointerstitium, and an infiltrating population of neutrophils and T and B lymphocytes. The presence of these insults in a *Rag1*^−/−^*Clr-f*^−/−^ background reveals that *Clr-f*^−/−^ mice are susceptible to a T and B lymphocyte-independent renal pathogenesis. Our data reveal a role for Clr-f in the maintenance of kidney immune and metabolic homeostasis.

## Introduction

Maintaining tolerance to harmless self-antigens can be a daunting task for renal immune cells, which are constantly exposed to generally innocuous low-molecular-mass proteins filtered from the circulation. This immune regulation must hold several layers of kidney defenses in check. Tubular epithelial cells, which regulate the exchange of ions, water, and small molecules between the glomerular filtrate and circulating blood, also possess the ability to recognize pathogenic or auto-antigens using a sophisticated repertoire of pattern recognition receptors (PRRs)^1^. Resident immune cells of the kidney, mainly dendritic cells (DCs), and a small population of macrophages and lymphocytes occupy the tubulointerstitial space surrounding the nephrons and also express a variety of PRRs^2–4^. In the context of kidney disease, renal damage often starts at the glomerulus and spreads to the immune cells of the interstitium. Glomerular injuries may cause a profusion of proinflammatory cytokines and leakage of the glomerular filtrate and autoantigens into the tubulointerstitium. This disruption results in the activation of infiltrating lymphocytes by DCs, which drives the additional production of inflammatory cytokines that lead to further tissue damage. While mechanisms for the ablation of autoreactive T cells in renal lymph nodes are proposed as a means of maintaining tolerance to self-ligands among intra-renal immune cells, our understanding of the role the kidney epithelium plays in maintaining homeostasis is less clear^5^.

One member of the C-type lectin-related (Clr) family of surface proteins, Clr-f (encoded by *Clec2h*), is strongly expressed in the mouse proximal tubular epithelium and the intestine^6,7^. Clr-f is one of seven members of the Clr protein family in mice (Clr-a/*Clec2e*, Clr-b/*Clec2d*, Clr-c/*Clec2f*, Clr-d/*Clec2g*, Clr-f/*Clec2h*, Clr-g/*Clec2i*, and Clr-h/*Clec2j*), which have distinct and overlapping tissue-specific patterns of expression^6^. The Clr proteins exhibit characteristic attributes of immune regulatory molecules^6^, such that the Clr proteins are commonly described in the functional paradigm as tissue-specific ligands of five C-type lectin receptors belonging to the NKR-P1 family [NKR-P1A (encoded by *Klrb1a*), NKR-P1C/*Klrb1c*, NKR-P1F/*Klrb1f*, NKR-P1D/*Klrb1d*, and NKR-P1G/*Klrb1g*] that mediate self- versus non-self-recognition and regulate innate immune responses^6,8–13^. In this receptor system paradigm, Clr-f is the reported self-ligand of the inhibitory NKR-P1G receptor^7^. In humans, the lectin-like transcript 1 (LLT1), encoded by *CLEC2D*, is the functional and structural ortholog of the mouse Clr proteins. LLT1 is expressed in a wide variety of cells and tissues including lymphocytes, DCs, osteoblasts^14–16^, and the gallbladder, digestive tract, lung pneumocytes, and renal tubular epithelium^17^. Similar to the receptor–ligand system in mice, LLT1 is recognized by the NKR-P1A lectin-like receptor, an ortholog of the mouse NKR-P1 proteins^18^.

Despite the significant levels of Clr-f expression in the kidney and intestinal epithelium, its function in these tissues is unclear. In the gut, Leibelt and co-workers showed that Clr-f is upregulated in intestinal epithelial cells after poly(I:C)-induced inflammation^7^, possibly to limit immune damage to the intestinal epithelium by intraepithelial lymphocytes that express the inhibitory NKR-P1G receptor. The tissue-specific expression of Clr-f suggests that the NKR-P1G:Clr-f recognition appears to regulate innate immune cells in a tissue-specific manner. However, the role of Clr-f in the renal epithelial cells, and its role in the regulation of innate immune responses is completely unknown in the kidney.

Here we explore the role of Clr-f in the kidney, using Clr-f-deficient mice. We show that mice lacking Clr-f develop pathological lesions involving the glomeruli and tubules. Additionally, we uncover an age-associated deposition of immune complexes in the glomeruli of Clr-f-deficient mice and the presence of renal infiltrating neutrophils, and T and B cells. Global transcript analysis of Clr-f-deficient kidneys revealed a dysregulation of lipid metabolic pathways and inflammatory pathways, which was mirrored in the pathological progression of Clr-f-deficient mice at old age, with mice exhibiting significant accumulation of abdominal fat tissue and lipids in the renal cortical interstitium. Our findings provide a kidney-protective role for Clr-f, which exhibits immunoregulatory activity over immune subsets, and may play a role in metabolic homeostasis in the kidney.

## Results

### Generation of Clr-f-deficient mice

**Figure 1:**
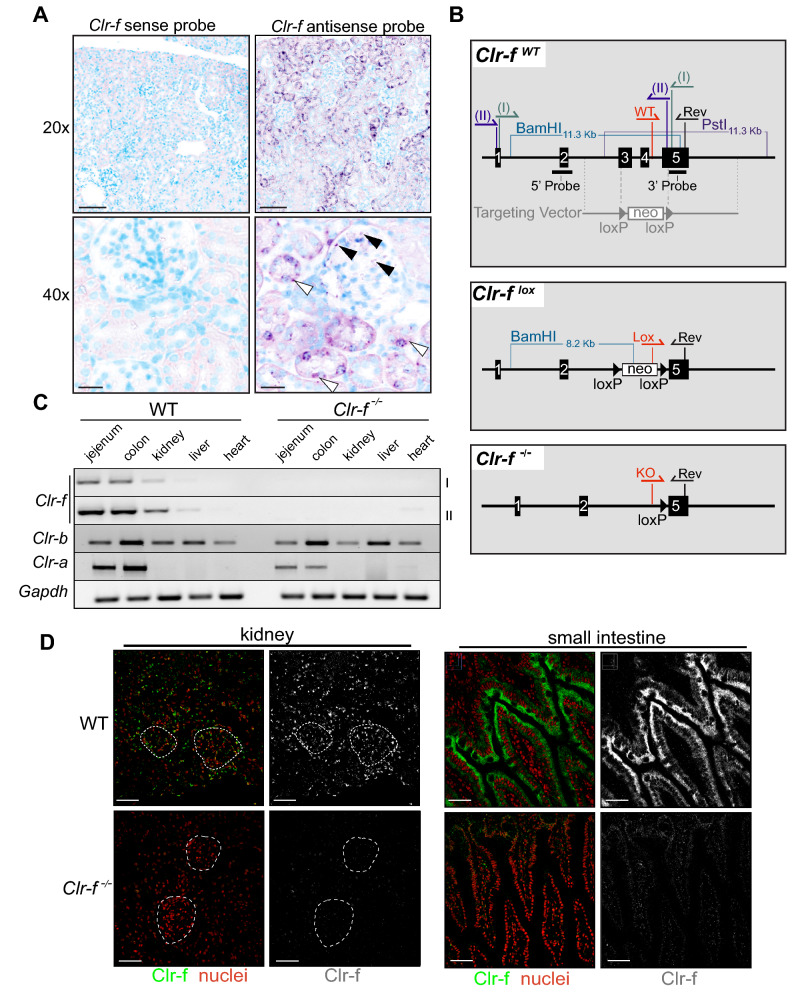
Generation of *Clr-f*^−/−^ mice by targeted *Clr-f* gene deletion. (**A**) In situ hybridization of full-length *Clr-f* DIG-labelled sense and antisense probes to WT kidney tissues. DIG staining of proximal tubular cells (white arrowheads), and podocytes (black arrowhead) indicated. Scale bars represent 100 μm in upper panels and 25 μm in lower panels. (**B**) Schematic of genetic strategy to ablate the *Clr-f* gene exons 3, 4, and part of 5 with targeting vector by homologous recombination. Southern blot analysis 5′ and 3′ probe targets, and *Bam*HI and *Pst*I restriction enzyme digestion sites, used to confirm ES cell clone targeting, are shown. Locations of PCR primer annealing sites for genotype validations performed in Fig. S1 are shown in red and black. Locations of *Clr-f* RT-PCR primers (I) and (II) annealing sites in exons 1 and 5 are shown. (**C**) RT-PCR analysis of the indicated transcript expression in various organs. Heart tissue-derived cDNA was used as a negative control for *Clr-f* transcript expression. Two primer sets were used to amplify the *Clr-f* transcript: the binding sites for PCR primers (I) or (II) are shown in the *Clr-f* ^WT^ panel B. Cropped gel images of separate gels are shown. Full-length gel images are shown in Fig. S3A. (**D**) IF of the renal cortex and small intestine of WT and *Clr-f*^−/−^ mice using the 10A6 antibody. Glomeruli are indicated in kidney IF panels, Clr-f staining is shown in green, nuclei are shown in red. Scale bars represent 40 μm.

Previous reports of the tissue-specific distribution of mouse Clr family members found low levels of Clr-f expression in the liver and very high expression in kidney and gut-epithelial tissues^6,7,19^. To resolve the cell-specific expression pattern of *Clr-f* in WT mouse kidneys, we probed kidney tissues with DIG-labeled *Clr-f-*specific RNA probes by in situ hybridization. We detected *Clr-f* transcript-specific staining in epithelial cells of the proximal convoluted tubules and less pronounced staining of the distal renal tubules (Fig. 1A). Signals were also detected in podocytes, but not in glomerular mesangial and endothelial cells, nor in the interstitium or lymphoid and vascular structures of the kidney, aligning with the *Clr-f* expression reported in whole kidney single-cell RNA sequencing^20^.

To explore the role of Clr-f in kidney health and function, we generated Clr-f-deficient mice (*Clr-f*^−/−^) by homologous recombination-mediated deletion of *Clr-f* exons 3, 4, and part of exon 5 (Fig. 1B and Fig. S1). RT-PCR of *Clr-f* gene expression in kidney, intestine, and liver cells of *Clr-f*^−/−^ mice confirmed a complete loss of detectable *Clr-f* transcript. We also confirmed that the most closely related Clr transcripts, the *Clr-b* and *Clr-a* transcripts, were still detectable (Fig. 1C). Immunofluorescent (IF) labeling of the kidney and small intestine of *Clr-f*^−/−^ mice confirmed the absence of Clr-f protein expression (Fig. 1D and Fig. S2A).

The *Clr-f* gene lies in apposition to the *Klrb1g* gene, which encodes NKR-P1G, the reported receptor for Clr-f. To assess whether the deletion in *Clr-f* alters the expression of *Nkrp1g*, we imaged NKR-P1G-labeled intestinal and splenic tissues of WT and *Clr-f*^−/−^ mice by IF (Fig. S2B,C) and measured NKR-P1G on CD3^+^ and NKp46^+^ splenocytes from WT and *Clr-f*^−/−^ mice (Fig. S2D,E). NKR-P1G-staining of gut and splenic tissues and isolated splenocytes of *Clr-f*^−/−^ mice were indistinguishable from that of WT mice, confirming that the *Clr-f* gene deletion does not alter NKR-P1G protein expression.

### Kidney tubular and glomerular lesions in Clr-f-deficient mice

**Figure 2:**
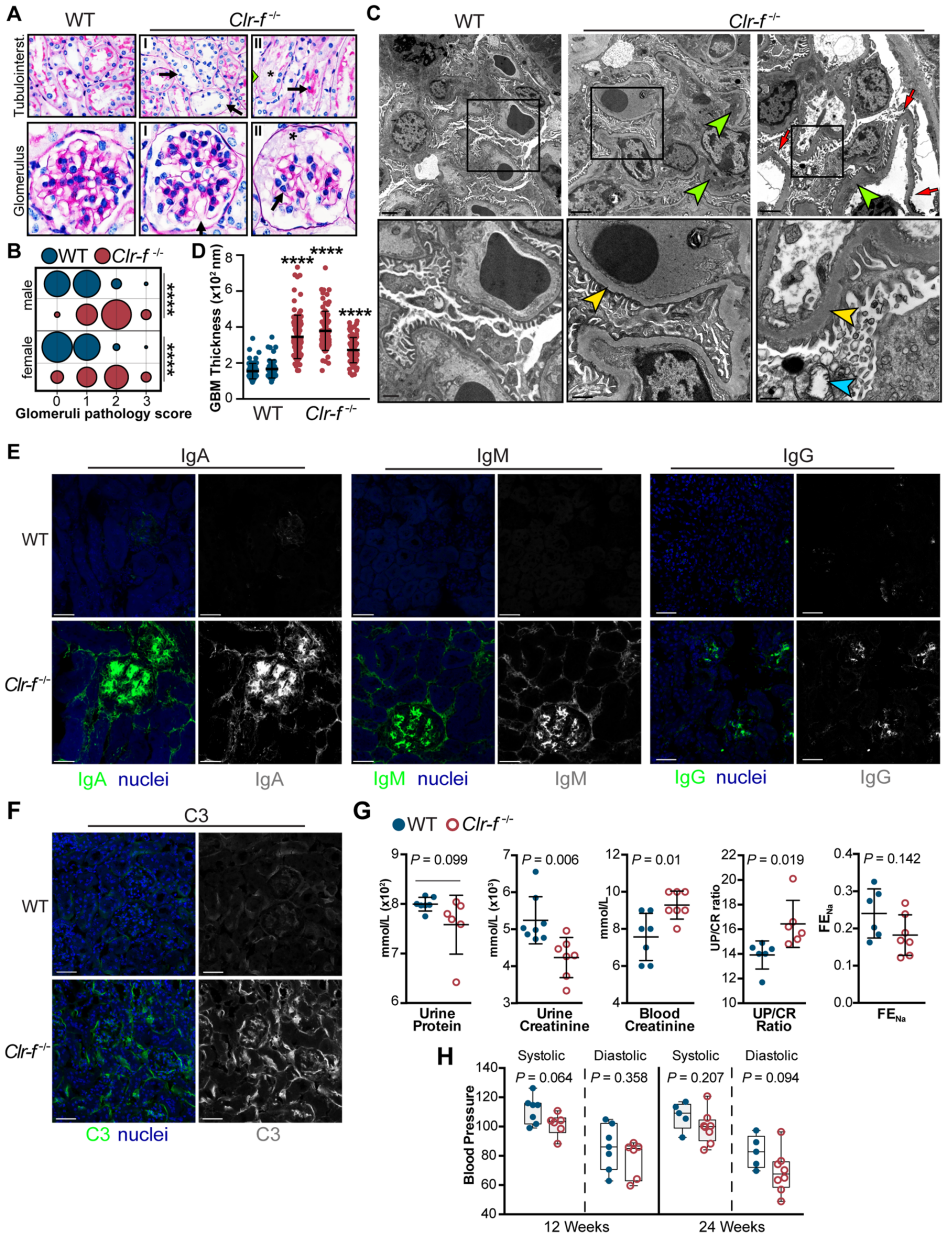
Clr-f-deficient mice exhibit kidney pathology and altered kidney function. (**A**) Renal tissue sections from 12-week-old WT and *Clr-f*^−/−^ mice (n = 4) stained with PAS. Upper panels show the appearance of lesions in proximal and distal tubules of *Clr-f*^−/−^ mice with severity ranging from minimal epithelial disturbance (not shown), (I) flattened tubular epithelium with numerous cytoplasmic vacuoles (arrows), nuclear apical displacement, and (II) moderate and aggravated lesions with the disappearance of tubular epithelium. The green arrowhead in the far right panel (II) indicates an area of cell loss where the tubular basement membrane is covered by a thin layer of cytoplasm. Arrows indicate detached cells in tubular lumina and the asterisk marks an area of accumulated necrotic debris and loss of epithelial cell brush border. Lower panels show glomerular sections from 12-week-old WT and *Clr-f*^−/−^ mice (n = 4) stained with PAS. *Clr-f*^−/−^ mice exhibit a varying extent of glomerular mesangiolysis with (I) primary phase and (II) fragmentation and pronounced disruption of glomeruli. Arrows indicate capillary aneurysms (arrow). An asterisk indicates necrotizing cellular debris and plasma in Bowman’s spaces. (**B**) Pathology scoring of the observed glomerular lesions. Scores are based on the level of disappearance of the glomerulus: 0: < 10%, 1: 10–30%, 2: 30–60%, 3: > 60%. The bubble area represents the proportion of total counts (****P ≤ 0.0001). (**C**) Transmission electron micrographs demonstrate severe podocyte destruction and podocyte foot process effacement in *Clr-f*^−/−^ mice (red arrows). The inset boxes in the upper panels are shown in the lower panels. Yellow arrowheads indicate GBM thickening, green arrowheads indicate subendothelial electron-dense deposits, blue arrowhead indicates mitochondrial cristolysis. Upper panel scale bars represent 2 μm. The lower left and middle panel scale bars represent 800 nm and lower right panel scale bar represents 500 nm. (**D**) Measurements of GBM thickness from electron microscopy micrographs of WT (n = 2) mice and *Clr-f*^−/−^ (n = 3) mice. Each point represents a distinct GBM measurement. (****P ≤ 0.0001). (**E**) IF staining of WT and *Clr-f*^−/−^ glomerular sections for IgA, IgM, IgG deposition, and (**F**) C3 complement protein in 12-week-old mouse kidneys. Scale bars represent 40 μm. (**G**) Protein and creatinine measurements in urine and serum samples and urinary fractional excretion of sodium (FE_Na_) values from 12-week-old male *Clr-f*^−/−^ and WT mice. (**H**) Blood pressure measurement in *Clr-f*^−/−^ mice compared to their WT littermates at indicated ages. Mean and standard deviation are shown.

The initial examination of *Clr-f*^−/−^ kidney sections revealed several alterations in the renal cortex. To evaluate histopathologic lesions, PAS-stained kidney sections from four 12-week-old WT and *Clr-f*^−/−^ mice were assessed in a blinded fashion (Fig. 2A). A variable presence of proximal and distal tubular lesions was observed in *Clr-f*^−/−^ kidneys which included, tubular epithelial cell flattening, nuclear displacement, and loss of brush borders (Fig. 2A; top panels). In rare instances, tubular necrosis was found in the form of luminal necrotic debris accumulation and complete epithelial loss in some of the examined areas. The *Clr-f*^−/−^ kidneys also exhibited glomerular pathologies (Fig. 2A; bottom panels), with areas of focal thickening of glomerular tufts, minimal periglomerular fibroblast proliferation associated with mild interstitial fibrosis, and more severe lesions with mesangiolysis, capillary aneurysms that progressed to pronounced disruption of glomeruli and a constant presence of necrotizing cellular debris in Bowman’s spaces. Glomerular pathology scoring revealed a significant number of glomerular lesions in Clr-f-deficient mice in comparison to WT littermates (Fig. 2B). Electron microscopy revealed severe podocyte damage, podocyte foot process effacement, and significant thickening of the glomerular basement membrane (GBM) (Fig. 2C,D).

To assess if kidney immunoglobulin deposits were present, which are characteristically associated with the pathologies observed, we stained renal cortex sections of WT and *Clr-f*^−/−^ kidneys for the presence of IgA, IgM, and IgG antibodies by IF (Fig. 2E). *Clr-f*^−/−^ kidneys show clear dominant IF staining of glomerular IgA deposits accompanied by pronounced IgM deposits and a lesser but consistent presence of IgG deposits. Likewise, we detected similar patterns of IgA, IgM, and IgG tubulointerstitial deposits in *Clr-f*^−/−^ kidneys. Complement protein C3 was also broadly detected in *Clr-f*^−/−^ kidneys but not in WT kidneys (Fig. 2F).

### Clr-f is required for proper kidney function

To evaluate the impact of *Clr-f*^−/−^ pathologies on kidney function, we analyzed blood and urine composition from 12-week-old male *Clr-f*^−/−^ and WT littermates. We found a modest, yet statistically significant, increase in the urine-protein-to-creatinine (UP/CR) ratio in *Clr-f*^−/−^ mice compared to WT mice, with increased serum creatinine, but similar urine protein levels compared to WT mice, as well as a similar fractional excretion of sodium (FE_Na_) (Fig. 2G). Given the tubular expression of Clr-f, we assessed if *Clr-f*^−/−^ mouse blood pressure in 12-week-old and 24-week-old *Clr-f*^−/−^ mice was affected. *Clr-f*^−/−^mice exhibited a marginal but non-statistically significant decrease in blood pressure compared to WT littermates (Fig. 2H). These results show that normal kidney filtration is altered in the absence of Clr-f, but Clr-f shows no significant roles in renal electrolyte homeostasis or blood pressure regulation.

### *Clr*-*f*^−/−^ kidney transcriptome shows a dysregulation in metabolism

**Figure 3:**
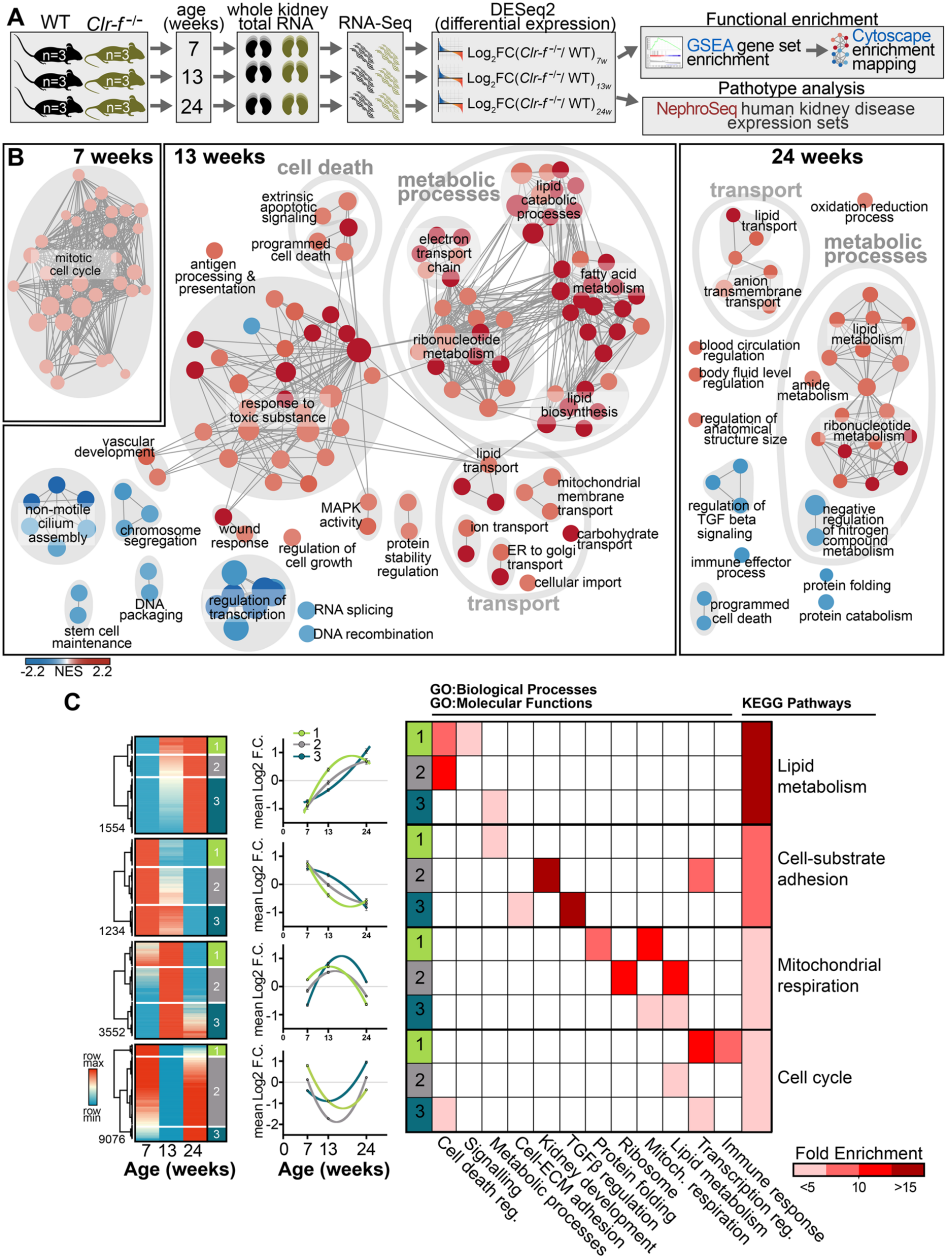
Gene expression profile of *Clr-f*^−/−^ kidneys reveals progressive dysregulation in metabolism and stress response. (**A**) Schematic of RNA sequencing pipeline. (**B**) Interaction network of gene set enrichment analysis for biological processes differentially enriched in *Clr-f*^−/−^ mice compared to WT at 7, 13, and 24 weeks. Blue nodes represent gene sets enriched with downregulated transcripts (Log_2_ FC < 0), and red nodes represent gene sets enriched with upregulated transcripts (Log_2_ FC > 0), according to the Normalized Enrichment Score (NES) color scale shown. Interaction map was constructed using Cytoscape V3.7.1 (**C**) Heatmaps represent *Clr-f*^−/−^ DEGs that belong to four dynamic patterns of expression: DEGs that increase with age, decrease with age, and DEGs that transiently increase or decrease at 13 weeks of age. Three sub-clusters of genes identified within each dynamic expression pattern represent three distinct variations in expression dynamics within the four aforementioned groups. The mean Log_2_FC of DEG sub-clusters within each dynamic pattern is plotted against time with lines of best fit shown (using least-squares second-order polynomial). Gene ontology categorization of GO:Biological Processes/GO:Molecular Function for genes belonging to the three sub-clusters of each dynamic pattern is indicated by red squares, with color intensity according to fold enrichment. Pathways enriched from KEGG pathways are shown for all genes belonging to each of the four dynamic patterns of expression. Heat map was constructed using Morpheus (https://software.broadinstitute.org/morpheus).

To investigate the underlying transcriptional processes responsible for *Clr-f*^−/−^ kidney pathophenotypes, we performed RNA-sequencing on kidneys of 7-, 13-, and 24-week-old WT and *Clr-f*^−/−^ mice. Differentially expressed genes (DEGs) in *Clr-f*^−/−^ mice compared to WT mice were investigated by functional enrichment analysis and transcriptional pathotype analysis (Fig. 3A). We mapped interaction networks of enriched biological processes from gene set enrichment analysis (GSEA) of DEGs identified at each age (Fig. 3B). Our analysis showed an enrichment of cell cycle-related genes upregulated in kidneys of 7-week-old *Clr-f*^−/−^ mice. At 13 weeks, metabolic processes, transport, cell death, and responses to toxic substances were enriched from DEGs with increased expression in *Clr-f*^−/−^ kidneys, while non-motile cilium assembly, DNA transcription, and processing were enriched from DEGs with decreased expression. Kidneys of 24-week-old *Clr-f*^−/−^ mice also exhibited an enrichment of metabolic processes, and transport among DEGs with increased expression, as well as processes that regulate blood circulation, while cell death, immunity, and Transforming growth factor-beta (TGFβ) signaling regulation processes were enriched from DEGs with decreased expression.

To capture age-associated processes that underlie *Clr-f*^−/−^ kidney defects, we analyzed genes belonging to four dynamic patterns of differential expression: DEGs that increase with age, decrease with age, or transiently increase or decrease at 13 weeks of age. Functional enrichment analysis of biological processes, molecular functions, and defined pathways among these groups revealed that DEGs with increasing expression from 7 to 24 weeks of age in *Clr-f*^−/−^ kidneys were overrepresented in lipid metabolic pathways, while DEGs related to cell-substrate adhesion decreased in expression from 7 to 24 weeks of age (Fig. 3C). DEGs with a transient increase in expression at 13 weeks of age were mostly associated with mitochondrial respiration, while DEGs with a transient decrease in expression mapped to cell cycle pathways. Within these four categories of dynamic expression were three sub-clusters that represent differential expression patterns in which the magnitude of change in expression between 7 and 13 weeks was either similar, higher, or lower than the degree of change between 13 and 24 weeks of age, respectively (Fig. 3C). The enriched biological processes and molecular functions from each sub-cluster of genes were largely distinct across the four main dynamic expression patterns identified but reflected overlapping or related cellular processes within each group. Overall, the transcriptional profile of *Clr-f*^−/−^ kidneys reveals a dynamic and evolving dysregulation of cellular metabolism, function, and homeostasis.

### *Clr*-*f*^−/−^ mice accumulate fat

**Figure 4:**
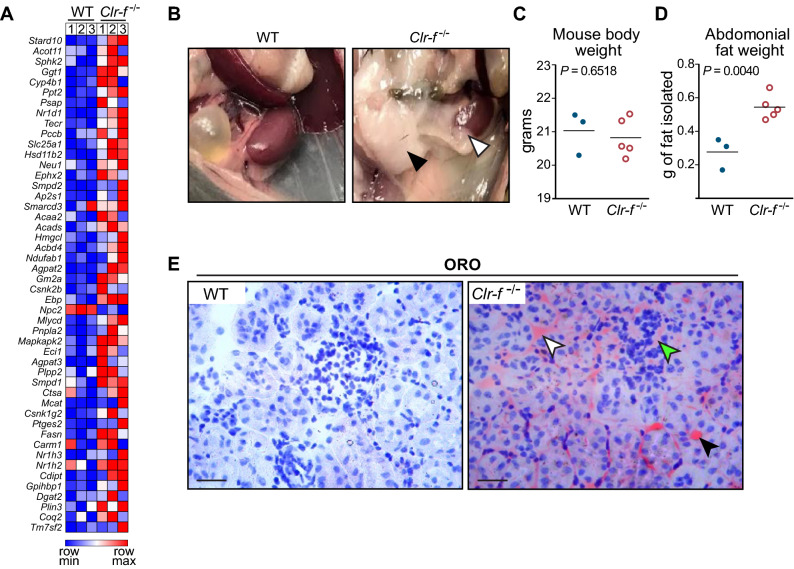
*Clr-f*^−/−^ mice accumulate abdominal, perirenal and tubulointerstitial fat. (**A**) Heatmap comparing FPKM values of genes with roles in lipid metabolism between 13-week-old WT and *Clr-f*^−/−^ mice. Heat map was constructed using Morpheus (https://software.broadinstitute.org/morpheus). (**B**) Exposed abdominal cavity of 24-week-old WT and *Clr-f*^−/−^ mice. Abdominal fat is indicated by the black arrowhead, and perirenal fat is indicated by a white arrowhead. (**C**) Total bodyweight of 24-week-old WT and *Clr-f*^−/−^ mice. (**D**) Weight measurements of total abdominal fat dissected from 24-week-old WT and *Clr-f*^−/−^ mice. Horizontal bars represent mean weight. (**E**) ORO lipid staining of kidney tissues from 24-week-old WT and *Clr-f*^−/−^ mice. White and black arrowheads indicate tubulointerstitial and luminal ORO staining, respectively, and the green arrowhead indicates glomerular ORO staining. Images are at 20 × magnification.

The identified age-associated increase in DEGs related to lipid metabolism led us to probe expression data of WT and *Clr-f*^−/−^ mice for known lipid-associated genes. Most of these gene transcripts were elevated in *Clr-f*^−/−^ mice (Fig. 4A) and aligned with increases in abdominal and perirenal adipose deposits in *Clr-f*^−/−^ mice at 24 weeks of age (Fig. 4B). Although *Clr-f*^−/−^ and WT mice exhibited similar body weights (Fig. 4C), the weight of abdominal adipose in *Clr-f*^−/−^ mice was significantly higher, indicating that adipose tissue accounted for an increased proportion of *Clr-f*^−/−^ mouse body weight (Fig. 4D). We also detected ectopic lipid accumulation within the kidneys of *Clr-f*^−/−^ mice, as evidenced by increased ORO staining of *Clr-f*^−/−^ mouse kidneys (Fig. 4E). As perirenal fat and the presence of interstitial fat in the kidney are associated with metabolic risk factors of CKD^21^, we hypothesized whether Clr-f is protective against etiologies of diabetes or related metabolic disorders associated with CKD.

### *Clr*-*f*^−/−^ mouse kidneys have transcriptional profile similarities and differences to human kidney disease

**Figure 5:**
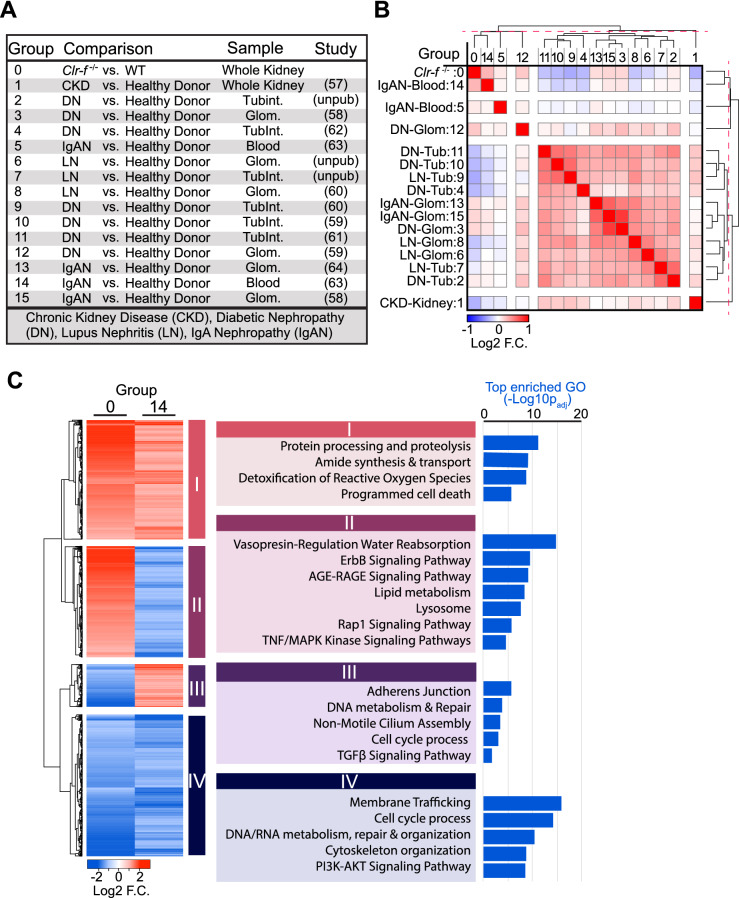
Comparison between transcriptional profiles of *Clr-f*^−/−^ mice and human CKDs. (**A**) Table of gene expression profile data sets of 15 human renal disorders vs. 13-week-old *Clr-f*^−/−^ mice. The reference source for the human expression profile data set sources is indicated by the Study column. (**B**) Similarity matrix showing hierarchical clustering of differential gene expression profiles in panel (**A**). Red-dashed line within dendrogram indicates the level of matrix cluster separation. (**C**) Comparison of 4531 DEGs (Log2 FC; *P*adj. < 0.05) of 13-week-old *Clr-f*^−/−^ mice (group 0) and IgAN patients. Gene set enrichment from 4 DEG clusters: (I) upregulated in both *Clr-f*^−/−^ mice and IgAN patients, (II) upregulated in *Clr-f*^−/−^ mice and downregulated IgAN patients, (III) downregulated in *Clr-f*^−/−^ mice, and upregulated IgAN patients, and (IV) downregulated in both *Clr-f*^−/−^ mice and IgAN patients. Heat maps were constructed using Morpheus (https://software.broadinstitute.org/morpheus).

Ectopic lipid accumulation in the kidney is associated with inflammation, fibrosis, mitochondrial dysfunction, and cell death^22,23^. To assess if *Clr-f*^−/−^ kidneys resemble defined kidney diseases, we compared the transcriptional profile of *Clr-f*^−/−^ kidneys to published expression profiles of kidney tissue and/or blood from human donors with CKD, diabetic nephropathy (DN), lupus nephritis (LN), or IgA nephropathy (IgAN) (Fig. 5A). *Clr-f*^−/−^ kidney transcriptional profiles clustered with blood from IgAN patients and glomerular tissue of DN patients (Fig. 5B). Comparison of *Clr-f*^−/−^ and IgAN patient expression profiles by functional enrichment analysis showed parallel increases in gene expression related to cellular reactive oxide responses, cell death, proteolysis, and amide metabolism, as well as shared decreases in gene expression associated with membrane trafficking processes, cytoskeleton organization, PI3K-AKT signaling, cell cycling, and DNA/RNA metabolism (Fig. 5C). Conversely, *Clr-f*^−/−^ kidney expression sets differed from IgAN, with increased expression of genes belonging to the AGE-RAGE, ErbB, TNF/MAPK, and Rap1 signaling pathways, and processes of vasopressin-regulated water reabsorption, and lysosome activity, and decreased expression of genes belonged to adherens junction, non-motile cilium assembly, cell cycling, DNA metabolism, and TGFβ-signaling. These findings suggest that similar to IgAN, cellular processes of growth control, homeostasis, and repair, are also dysregulated in *Clr-f*^−/−^ kidneys, while altered metabolism, stress-signaling, and epithelial structure and function are unique to *Clr-f*^−/−^ kidneys.

### Clr-f-deficient kidneys exhibit inflammation and immune cell infiltration

**Figure 6:**
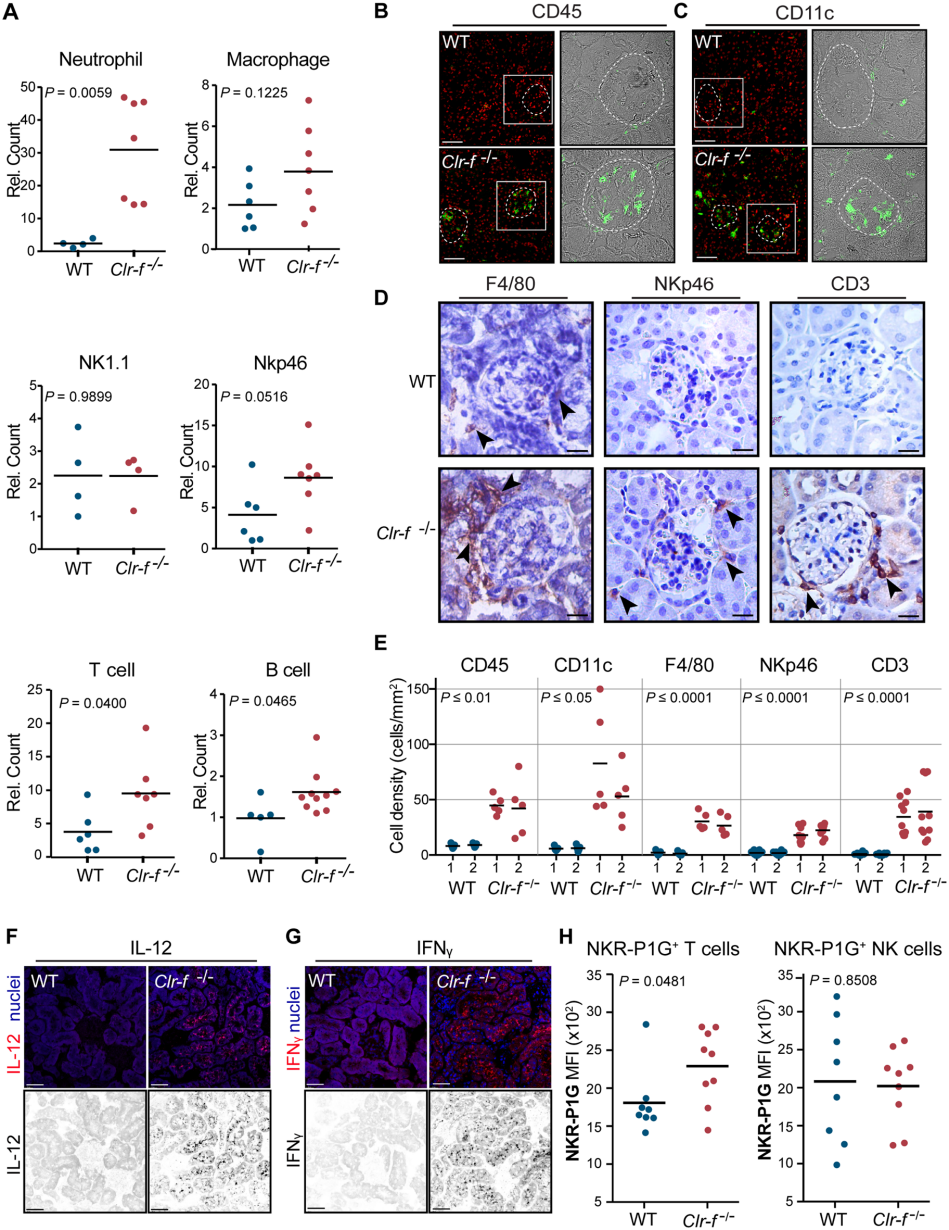
Presence of renal immune cells in kidney tissue in *Clr-f*^−/−^ mice. (**A**) Flow cytometry analysis of neutrophil, macrophage, NK1.1^+^ cell, NKp46^+^ cell, T cell, and B cell numbers per the whole kidney of *Clr-f*^−/−^ mice relative to WT mice at 12 weeks of age. Horizontal bars represent mean cell numbers. (**B**) IF staining of WT and *Clr-f*^−/−^ mouse kidneys with anti-CD45 antibody or (**C**) anti-CD11c antibody and DAPI nuclear staining (in red). Glomeruli are indicated by white-dashed lines. Brightfield panels of select glomeruli are shown with CD45^+^ or CD11c^+^ cells shown (in green). Scale bars represent 40 μm. (**D**) IHC staining of WT and *Clr-f*^−/−^ mouse kidneys with antibodies against F4/80, NKp46, and CD3. Black arrowheads indicate select IHC positive cells. Images are captured at 20 × magnification. Scale bars represent 40 μm. (**E**) Visual quantification of indicated immune cell type per field from kidney sections of two WT and two *Clr-f*^−/−^ mice. Each dot represents an individual field count and horizontal bars represent mean cell numbers. (**F**) IF staining of kidney sections for IL-12 and (**G**) IFNγ cytokines in WT and *Clr-f*^−/−^ mice. Scale bars represent 40 μm. (**H**) NKR-P1G mean-fluorescence intensity (MFI) on kidney TCRβ^+^ T cells and NK1.1^+^ NK cells measured by flow cytometry analysis. Horizontal bars represent mean cell numbers and error bars represent standard deviation.

Given the known immunomodulatory roles for Clr proteins, we examined the immune cell contribution to *Clr-f*^−/−^ kidney pathology. We analyzed the immune cell composition of 12-week-old WT and *Clr-f*^−/−^ mouse whole kidneys by flow cytometry. Our analysis revealed a significant increase in the number of neutrophils, T cells, and B cells, but no difference in renal NK cell, ILC or macrophage numbers (Fig. 6A). Analysis of the periglomerular distribution of immune cells within the kidney cortex of WT and *Clr-f*^−/−^ kidneys by IF, we observed glomerular accumulations of CD45^+^ cells (Fig. 6B). Labeling of CD11c, F4/80, NKp46, and CD3, markers indicative of DCs, macrophages, NK/ILCs, and T cells respectively, revealed an intraglomerular accumulation of CD11c^+^ cells (Fig. 6C), and periglomerular accumulations of F4/80^+^, NKp46^+^, and CD3^+^ cells in *Clr-f*^−/−^ kidneys (Fig. 6D). Visual quantification of immune cell populations detected at the glomeruli confirmed a significant increase in the presence of each of these immune cell populations in kidneys of *Clr-f*^−/−^ mice compared to WT mice (Fi.g 6E). Immune infiltrate in autoimmune kidney disease is enhanced by the tubular secretion of IL-12 prompting injury by IFNγ secreting cells^24–26^. We, therefore, examined WT and *Clr-f*^−/−^ kidneys for the presence of these cytokines and observed staining of both IL-12 and IFNγ in cells of the *Clr-f*^−/−^ kidney tubulointerstitium (Fig. 6F,G).

Since Clr-f is posited to be a signal of cell health to NKR-P1G-expressing cells, we hypothesized that the renal immune infiltration in *Clr-f*^−/−^ mice is partly due to an increased presence of NKR-P1G-expressing lymphocytes in the kidney. Analysis of NKR-P1G expression on kidney TCRβ^+^ T cells and NK1.1^+^ NK cells in WT and *Clr-f*^−/−^ mice by flow cytometry showed no difference in the mean fluorescence intensity of NKR-P1G staining on renal NK cells, but an increase in NKR-P1G staining of renal T cells in *Clr-f*^−/−^ mice (Fig. 6H).

### Clr-f deficiency results in T- and B-independent autoimmunity and pathogenesis

**Figure 7:**
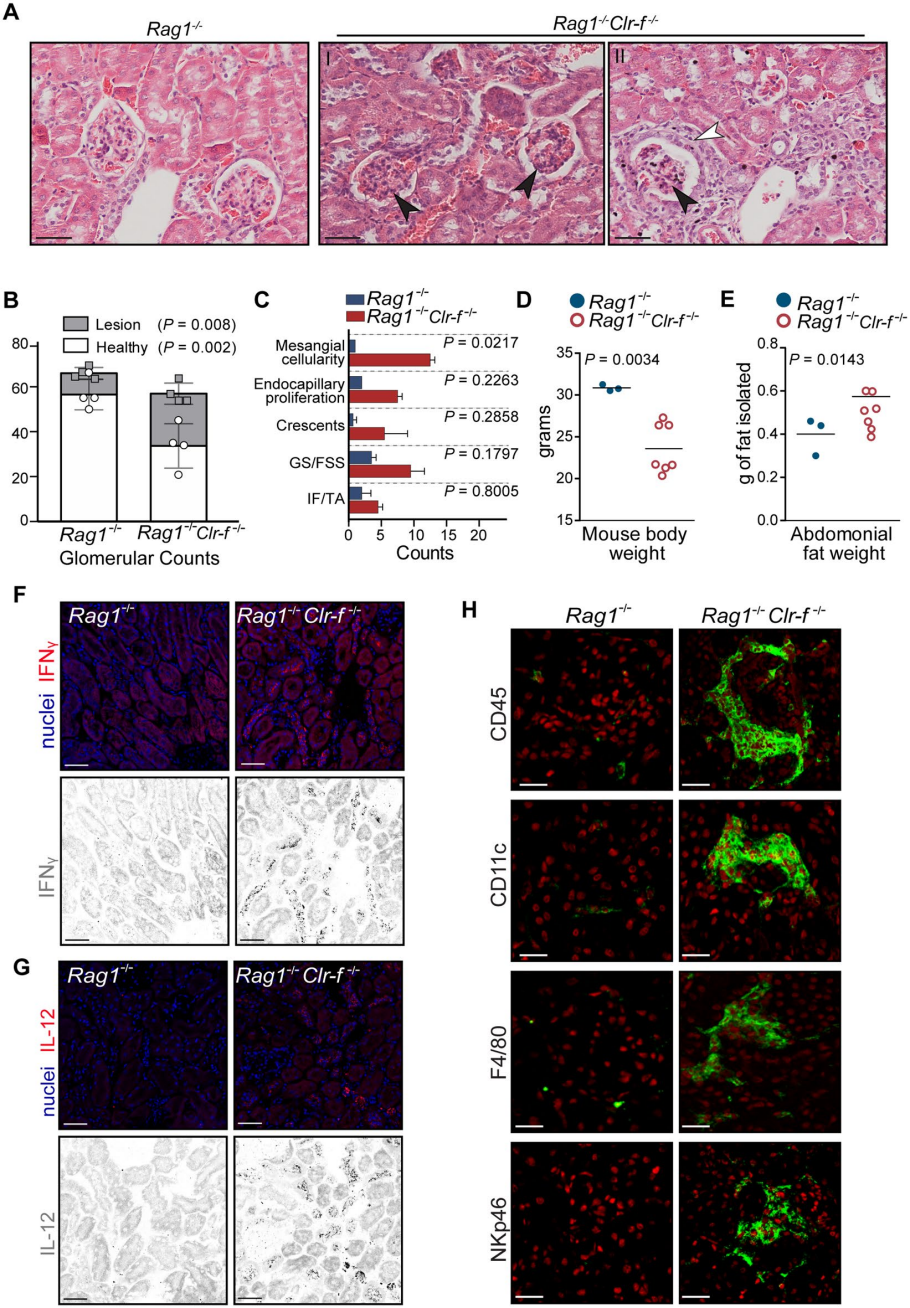
Presence of T and B cell-independent kidney pathology in *Clr-f*^−/−^ mice. (**A**) Light microscopy images of H & E-stained kidney section from *Rag1*^−/−^ and *Rag1*^−/−^*Clr-f*^−/−^ mice. *Rag1*^−/−^*Clr-f*^−/−^ kidneys shown in (I and II) exhibit glomerular hypercellularity (black arrowheads) and *Rag1*^−/−^*Clr-f*^−/−^ kidney shown in (II) exhibit fibrosis (white arrowheads). Scale bars represent 50 μm. (**B**) Scoring of glomerular lesions in *Rag1*^−/−^ and *Rag1*^−/−^*Clr-f*^−/−^ H&E-stained kidney sections. Each point represents glomeruli number per kidney that exhibited no overt pathology (circles) or exhibited pathological lesions (squares). Stacked columns indicated score means and error bars represent standard deviation. (**C**) Scoring of mesangial cellularity, endocapillary proliferation, crescents, glomerulosclerosis, or global or focal segmental sclerosis (GS/FSS), and interstitial fibrosis and tubular atrophy (IF/TA). Counts represent the average number of lesions per kidney section (n = 2 mice). Error bars represent standard deviations. (**D**) Measurement of total body weight for indicated mouse genotypes. (**E**) Measurement of total abdominal fat weight dissected from mice of the indicated mouse genotypes. Horizontal bars represent the mean weight. (**F**) IF staining of kidney sections for IFNγ (**G**) and IL-12 cytokines in *Rag1*^−/−^ and *Rag1*^−/−^*Clr-f*^−/−^ mice. Scale bars represent 40 μm. (**H**) IF staining of *Rag1*^−/−^ and *Rag1*^−/−^*Clr-f*^−/−^ mice with antibodies against CD45, CD11c, NKp46, or F4/80 (green) and nuclear staining with DAPI (in red). Scale bars represent 10 μm.

To clarify the immune contribution to the kidney pathology of *Clr-f*^−/−^ mice, we examined *Clr-f*^−/−^ mice that were also deficient in T- and B-cells, using the Rag1-deficient mouse strain (*Rag1*^−/−^*Clr-f*^−/−^). As *Rag1*^−/−^*Clr-f*^−/−^ mice lack antibodies, we could evaluate Clr-f-associated pathogenesis in the absence of immunoglobulin deposition. Analysis of the *Rag1*^−/−^*Clr-f*^−/−^ kidneys revealed glomerular lesions indicative of mild proliferative glomerulopathy and areas of glomerular fibrosis (Fig. 7A). Glomerular pathology scoring showed a significantly higher number of glomeruli with lesions in *Rag1*^−/−^*Clr-f*^−/−^ compared to *Rag1*^−/−^ mice (Fig. 7B). Scoring of *Rag1*^−/−^*Clr-f*^−/−^ kidney sections identified increases in endocapillary proliferation, crescents, glomerulosclerosis or focal segmental sclerosis, and interstitial fibrosis and tubular atrophy, with a significant increase in the presence of mesangial cellularity (Fig. 7C).

To assess T and B cell contribution to the lipid accumulation observed in *Clr-f*^−/−^mice, we measured total body weight and abdominal adipose weight in *Rag1*^−/−^*Clr-f*^−/−^ relative to *Rag1*^−/−^ mice. The *Rag1*^−/−^*Clr-f*^−/−^ body weight was significantly lower than *Rag1*^−/−^ controls (Fig. 7D), while abdominal lipid weight was significantly increased (Fig. 7E), albeit to a lesser extent than that observed in *Clr-f*^−/−^ mice compared to WT mice (174 mg of fat gained versus 274 mg on average).

Finally, to investigate the immune contributors to these kidney defects, we examined *Rag1*^−/−^*Clr-f*^−/−^ kidneys for the presence of IL-12 and IFNγ and renal infiltrating immune cells. IF analysis of *Rag1*^−/−^*Clr-f*^−/−^ kidneys revealed an increased presence of IL-12 and IFNγ (Fig. 7F,G). We also observed an extensive periglomerular accumulation of CD45^+^ cells in *Rag1*^−/−^*Clr-f*^−/−^ kidneys (Fig. 7H), which was found to consist of CD11c^+^, F4/80^+^, and Nkp46^+^ cells. These findings suggest that T and B cell-independent mechanisms are the primary mediators of renal pathology in mice deficient for Clr-f.

## Discussion

Inflammation and immune cell-mediated damage play a leading role in the development and progression of renal injury and dysfunction. In this study, we report a requirement for Clr-f in the maintenance of kidney health and function as an intrinsic homeostatic control against immune- and metabolic-mediated renal damage. Our findings show immune cell infiltration, and immunoglobulin and complement protein deposition in kidneys of Clr-f-deficient mice. These immune features of Clr-f-deficient mice are paired with the age-associated appearance of tubular and glomerular lesions, ectopic lipid accumulation, and the dysregulation of numerous transcriptional processes.

The Clr proteins encoded within the NKC among their genetically linked NKR-P1 receptors have been the focus of study as non-redundant MHC class I-independent mediators of missing-self recognition by NKR-P1 expressing immune cells^9,27–31^. The increased expression of select Clr proteins in response to cell stress is also reported to functionally restrain the immune responses of tissue-resident immune cells^7,12,32^. Accordingly, intestinal Clr-f was posited to contribute to immune tolerance within the otherwise highly immunogenic gut environment through its interaction with the inhibitory NKR-P1G receptor-expressing intraepithelial lymphocytes^7^. Work done by Leibelt et al. showed that mice challenged with poly(I:C) exhibited increased Clr-f expression and that Clr-f/NKR-P1G interactions in vitro are strongly inhibitory for NKR-P1G expressing cells. Together these findings can be interpreted as a potential mechanism by which Clr-f may prevent the reactivity of intestinal intraepithelial NKR-P1G positive subsets towards the epithelial barrier that is constantly provoked by microbial stimuli^7^.

The generation of Clr-f-deficient mice provided us the first opportunity to assess the functional implications of Clr-f expression in vivo and permitted us to investigate the potential immune homeostatic roles of Clr-f in the kidney. The kidney damage observed in *Clr-f*^−/−^ mice aligns with the model that the downregulation of Clr-f serves as an indicator of cell stress and injury that releases constraints on NKR-P1G-expressing effector cells. The increase in NKR-P1G on T cells in the kidneys of *Clr-f*^−/−^ mice suggests that either NKR-P1G expression is increased among kidney-resident T cells in the absence of Clr-f, or that the kidneys of *Clr-f*^−/−^ mice are subjected to an infiltration of NKR-P1G-expressing T cells. The absence of a detectable change in the presence or distribution of NKR-P1G-expressing cells in the gut of *Clr-f*^−/−^ mice might indicate that the functional consequences of the NKR-P1G:Clr-f interaction in the gut are limited to the control of immune reactivity in the context of infection rather than immune tolerance. Alternatively, redundant interactions may also control the activity of NKR-P1G-expressing cells. This is supported by findings that NKR-P1G binds not only to Clr-f but also to Clr-d and Clr-g^33^, the latter of which is moderately expressed in the tissues of both the kidney and the gut^6^. While our findings reveal a requirement for Clr-f to protect against autoimmune kidney damage, the NKR-P1G-mediated role in the autoimmune kidney pathologies of *Clr-f*^−/−^ mice is incomplete. Notably, although there is an increase in NKR-P1G-expressing T cells in the kidney of *Clr-f*^−/−^mice, the difference is underwhelming. It is possible that an alternative receptor participates in the surveillance of *Clr-f* in the kidney. To fully address this axis of control in kidney homeostasis, further investigations are needed.

Immune-mediated kidney damage is typified by an infiltration of inflammatory cells into the interstitial space or to the glomerulus^34^. The renal accumulations of monocytes, B and T cells, anti-glomerular antibodies, elevated IL-12 and IFNγ cytokines, and various glomerular pathologies in *Clr-f*^−/−^ mice closely resemble the autoimmune landscape of the LN mouse models (ie, MRL-*Fas*^*lpr*^)^35–37^. Although *Clr-f*^−/−^ kidneys exhibit IgA-dominant mesangial deposits with associated IgM and IgG deposition, and the clustering of *Clr-f*^−/−^ and IgAN transcriptional profiles, it is likely premature to suggest that these phenotypes are indicative of a type of CKD with IgAN^38^. Rather, these injuries, along with the presence of mesangiolysis, GBM thickening, and podocyte death in *Clr-f*^−/−^ kidneys as well as tubulointerstitial changes, fat accumulation, and the significant dysregulation in metabolic pathways are elements common to a variety of pathologies derived from metabolic defects exacerbated by the immune system^37,39,40^. Regardless of the underlying cause or disease type of the *Clr-f*^−/−^ mouse kidney disfunction, Clr-f appears to play a mitigating role against the development of autoimmunity with a clear glomerulonephritis-related pathology.

Our examination of the *Rag1*^−/−^*Clr-f*^−/−^ mice indicates a prominent contribution of T and B cell-independent mediators to the autoimmune responses of *Clr-f*^−/−^ mice. In particular, the accumulation of periglomerular CD11c^+^ cells, F4/80^+^ cells, and NKp46^+^ cells was even more pronounced in the absence of T and B cells and correlated with a significant presence of glomerular damage. This suggests that while T and B cells can contribute to kidney damaging inflammation and autoantibody complexes, in *Clr-f*^−/−^ mice these may not be central determinants of pathology, but either secondary contributors to the inflammatory activity of other immune cells or potentially serving an immunoregulatory role in mice that lack Clr-f. This contrasts evidence from the ddY mouse model of IgAN which shows a strong Th1 polarized response, with IFNγ-producing T cells increasing with age and the progression of glomerular injury^41^. The renal infiltration of B cells in Clr-f-deficient mice has also been shown in mouse models of SLE^42^. To date, it is unclear whether B cells in the kidney contribute to the damaging inflammation in CKD or play more regulatory roles^43^. Recent evidence of CKD in elderly patients found that decreases in B cell populations were associated with the progression of kidney disease^44^. Evidence from Both T and B cells have been shown to have immune-suppressive activity in autoimmune kidney diseases^45,46^, and therefore the extent to which they contribute or regulate the pathologies of *Clr-f*^−/−^ mice requires further investigation.

Our findings together show a requirement for Clr-f expression in the tubular epithelium of the proximal convoluted tubules, and podocytes, and/or the intestinal epithelium. Loss of Clr-f expression leads to a pronounced autoimmune and inflammatory cascade that leads to kidney damage and associated adiposity. The immune responses elicited by the loss of Clr-f appear to be T and B cell-independent. Overall, a functional role for Clr-f in the kidney can be envisioned as an inhibitory molecule for surveillant effector immune cells in the kidney as posited in the gut, or as an intrinsic regulator of tubular cell function.

## Materials and methods

### Mice

All mice were maintained in a dedicated pathogen-free environment. C57BL/6 (WT) and B6.129S7- *Rag1*^*tm1Mom*^ /J (*Rag1*^−/−^) mice were acquired from the Jackson Laboratory (Bar Harbor, ME, USA). Mice carrying the combined null mutations in *Rag1* and *Clr-f* (*Rag1*^−/−^;*Clr*-*f*^−/−^) were generated by crossing Clr-f-deficient (*Clr*-*f*^−/−^) mice with (*Rag1*^−/−^) mice. All breeding and manipulations performed on animals were in accordance with university guidelines and approved by the Dalhousie University Animal Ethics Committee and the Institutional Animal Care and Use Committee (IACUC) of the University of Ottawa. All animal study designs, experiments and reporting were conducted in accordance with the ARRIVE guidelines.

### Generation of Clr-f-deficient mice

Clr-f-deficient (*Clr*-*f*^−/−^) mice were generated by homologous recombination of a targeting construct containing a floxed phosphoglycerate kinase (PGK)–neomycin cassette and genomic sequence spanning exons 3, 4, and part of exon 5 of the *Clr-f* gene. The complete targeting construct was confirmed by sequencing at the Ottawa Hospitals Research Institute (OHRI) before electroporation into C57BL/6 Bruce-4 embryonic stem (ES) cells. ES clone selection by G418-resistance was performed by the IRCM (Montreal Clinical Research Institute) Transgenic Core Facility (Montreal, QC, Canada). Meanwhile, the 5′ and 3′ *Clr-f* probes were tested in a restriction fragment length polymorphism (RFLP) analysis using thymic genomic DNA from a WT B6 mouse, which was digested with *Bam*HI and *Pst*I, respectively. Digested DNA fragments were resolved on 1% agarose gel and transferred to nylon membrane blots. *Clr-f* cDNA probes were labeled with radioactive ^32^P-dCTP (Perkin Elmer, Guelph, ON, Canada) using the NEB lot kit (New England Biolabs, USA). Blots were probed with ^32^P-labelled *Clr-f* cDNA probes in hybridization solution (10% (w/v) dextran sulfate; 1 M NaCl; 1% SDS). Hybridization was carried out overnight at 65 °C and post-hybridization washes were performed with a solution containing 2 × SSC and 1% SDS pre-warmed to 65 °C. The blots were exposed to photographic films to reveal the hybridization signals (Figs. S1A, S3B). The membranes were stripped with 0.2% SSC/0.2% SDS for 30 min at 85 °C between different hybridizations. Neomycin-resistant clones were screened by Southern blot analysis, and a targeting efficiency of ~ 15% was observed. Selected ES clones were microinjected into blastocysts at the IRCM Microinjection Service Facility to generate the chimeric *Clr-f*^lox^ founder mice. The mice were screened by Southern blot analysis and were then bred with B6 females to produce *Clr-f*^wt/lox^ heterozygous mice. Heterozygous mice were interbred to obtain *Clr-f*^lox/lox^ mice. To remove the neomycin cassette, *Clr-f*^lox/lox^ mice were bred with homozygous CMV-cre transgenic mice on a B6 background (Jackson Laboratory). The resulting double heterozygous CMV-Cre^Tg/0^;*Clr-f*^*−/*+^ mice were interbred to produce *Clr-f*^−/−^ mice that lacked the CMV-cre transgene. Mice were genotyped using specific primers (Table 1, Fig. S1B,C, and Fig. S3C,D). PCR amplification was performed using the AccuStart II Taq DNA polymerase (Quanta Biosciences, Gaithersburg, MD, USA). PCR mixture was prepared following the manufacturer’s instructions. Phenol/Chloroform extracted ear DNA was used as templates. The following PCR cycling parameters were used: 30 amplification cycles of 94 °C for 40 s, 60 °C for 45 s, 72 °C for 60 s. WT and *Clr*-*f*^−/−^ littermates were used in all experiments unless otherwise indicated.

**Table 1:** Primer List.

Primer name	Forward primer (5′–3′)	Reverse primer (5′–3′)
KO	TCCTGGAAGGCTCTGGATTG	GACCTCGGAATGCCACTTGTC
WT	CAGTGAGGTCAGATGTGGCAG	GACCTCGGAATGCCACTTGTC
Lox	GACAATAGCAGGCATGCTG	GACCTCGGAATGCCACTTGTC
*Clr-f* (I)	TTGAAACGAGTTCCATGGGC	GGTCATAGAGCATCTGATTG
*Clr-f* (II)	ATGAATGCTGCAAAGGTTG	TCACACATGCCTTGGTAC
*Clr-b*	ACTCAGCTCCTCAGCTCTGA	GGCTAAAAAGCGTCTCTTGG
*Clr-a*	CAAAGGTTGAAGAGGCTTCC	TCACGCATGCTTTGGCACAT
*Gapdh*	ACTCACGGCAAATTCAACGGC	ATCACAAACATGGGGGCATCG

### In situ hybridization

In situ hybridization analyses of *Clr*-*f* were achieved by cloning the full-length sense-strand CDS and *Clr-f*-spanning antisense strand into a pGEM-T Easy vector as described previously. Digoxigenin-labeled antisense and sense RNA probes were transcribed from linearized plasmids encoding the *Clr-f* gene with T7 polymerase using DIG RNA labeling mix (Roche, Basel, Switzerland). Tissues were collected from adult B6 mice and were fixed either in 10% formalin at RT or in 4% paraformaldehyde at 4 °C with gentle shaking for up to 24 h. Tissues were then embedded in paraffin and 4 µm sections were prepared on glass slides. Tissue slides were deparaffinated by treatment with 0.2 N HCl for 15 min and 30 µg/mL of proteinase K at 37 °C for 20 min. Tissue was fixed in 4% PFA for 10 min followed by treatment with 0.25% acetic anhydride in 0.1 M triethanolamine two times for 5 min each. Slides were then pre-hybridized with hybridization solution (50% (v/v) formamide/5 × SSC, pH 4.5; 2% (w/v) blocking powder (Roche); 0.05% (w/v) CHAPS; 5 mM EDTA; 50 µg/mL heparin; 1 µg/mL yeast RNA) in a 58 °C oven for at least 1 h, and then incubated with hybridization solution containing 500 ng/mL digoxigenin-labeled probe overnight at 58 °C. Post-hybridization washes were performed with 50% formamide/2 × SSC, pH 4.5 at 55 °C 3 × for 20 min each, and slides were stained with sheep anti-digoxigenin alkaline phosphatase-conjugated antibody at 1:1000 dilution in blocking solution (Roche). Color development was performed by adding nitro blue tetrazolium/5-Bromo, 4-chloro, 3-indoylphosphate (NBT/BCIP, Roche) substrates to the slides. Slides were kept in the dark at RT until optimal signals appeared. Control labeling was performed using a sense-strand probe and competitive binding studies with sense and antisense probe confirmed specificity. Slides were then counterstained with light-green or methyl green and visualized under a light microscope.

### RT-PCR

Kidney tissues from age- and sex-matched WT and *Clr*-*f*^−/−^ mice were excised, rinsed with PBS, and frozen at -80 °C. RNA was isolated from frozen tissues using RiboZol RNA extraction reagent (AMRESCO Inc., West Chester, PA, USA) following the manufacturer’s instructions. Approximately 1 μg of RNA was transcribed into cDNA using Verso cDNA kit (Thermo Scientific, Waltham, MA, USA). PCR amplification was performed on 1 μL of cDNA product using specific primers (Table 1). PCR conditions used were 94 °C for 30 s, 58 °C for 30 s, 72 °C for 60 s, and 35 amplification cycles. PCR products were visualized on 1% agarose gel stained with ethidium bromide (Fig. 1C, Fig. S3A).

### Immunohistochemistry

Kidney tissues of age- and sex-matched WT and *Clr*-*f*^−/−^ mice were deparaffinized by three 5 min incubations in xylene, two 5 min incubations in 100% ethanol, followed by 90% ethanol, then 80% ethanol, and finally 70% ethanol. Slides were rehydrated by washing in distilled water (20 dips), and heat-induced epitope retrieval was performed using a pressure cooker in citrate buffer (pH 6.0). Endogenous peroxidases were blocked with 3% hydrogen peroxide in sterile water for 10 min, and proteins were blocked using Background Sniper (Biocare Medical, Pacheco, CA, USA). Sections were then incubated with rat anti-mouse monoclonal anti-Clr-f antibodies^7^ at 4 °C overnight. The next day, the slides were rinsed with 0.1 M PBS and incubated for 1 h in secondary antibodies. Staining was developed using a Goat-on-Rodent HRP-Polymer kit (Biocare Medical) and Betazoid DAB Buffer (Biocare Medical), followed by hematoxylin counter-staining. The same process was used to stain renal tissue section for IHC labeling of NKp46^+^ and CD3^+^ cells, using anti-NKp46 (eBioscience, Santa Clara, CA, USA, clone 29A1.4, CAT#48-3351-82) and anti-CD3 (BioLegend CAT#100,327) respectively as primary antibodies. IHC labeling of F4/80 was performed on frozen kidney sections using an anti-F4/80 rat monoclonal BM8 antibody. A goat anti-rat IgG H&L HRP-conjugated secondary antibody (Abcam, Cambridge, UK CAT#ab97057) was used as above and IHCs were developed with, a horseradish peroxidase (HRP) substrate was used DAB Substrate Kit (Abcam, CAT#ab64238).

### Immunofluorescence

The kidney and/or intestinal tissues of age- and sex-matched WT and *Clr*-*f*^−/−^ mice were cryopreserved in 30% sucrose at − 80 °C in Tissue-Tek optimal cutting temperature compound (Fisher Scientific, Pittsburgh, PA, USA). 20 μm cryostat sections were fixed with acetone in a fume hood at room temperature for 16 min and airdried for at least 30 min, after washing in 0.01 M PBS, pH 7.4. Nonspecific binding was blocked with 10% donkey serum. Sections were incubated overnight at 4 °C with the primary antibodies: anti-Clr-f^7^, anti-NKR-P1G (a rat anti-mouse monoclonal antibody described previously^7^), anti-IgA (goat anti-mouse IgA alpha chain (Abcam, Cambridge, UK CAT#ab97235), anti-IgG-PE (BioLegend, San Diego, CA, USA CAT#405324, anti-IgM-FITC (Life Technologies, Carlsbad, CA, USA CAT#A21042), Complement C3 Antibody (11H9) (Novus Biologicals, Littleton, CO, USA Cat# NB200-540), anti-IFNγ-PE (BD-Biosciences, Franklin Lakes, NJ, USA CAT#554411), anti-IL-12-PE (BD-Pharmingen CAT# 554479), anti-CD45-FITC (BioLegend CAT#103108), anti-CD11c-FITC (eBioscience CAT#11-0114-85), anti-F4/80-APC (BioLegend CAT#123116), and anti-NKp46-eFluor 450 (eBioscience, CAT#48-3351-82) at 1:100 concentration in 5% normal serum in a humidified chamber. Secondary antibody labeling of anti-Clr-f and anti-NKR-P1G antibodies using FITC-conjugated polyclonal antibodies was performed at RT for 1 h incubation. Samples were DAPI nuclear stained at RT for 5 min, washed with PBS, and mounted on glass slides with one drop of VECTASHIELD PLUS antifade mounting media (Vector Laboratories, San Francisco, CA, USA) and covered with a glass coverslip. Slides were examined using a Zeiss LSM 510 laser scanning confocal microscope.

### Histology and pathology scoring

Kidney sections of age- and sex-matched WT, *Clr*-*f*^−/−^, *Rag1*^−/−^ and *Rag1*^−/−^*Clr*-*f*^−/−^ mice (3 μm) were Periodic acid-Schiff (PAS) stained. Renal tissue was assessed for mesangial cellularity (defined as 4 or more nuclei per mesangial area), endocapillary proliferation, crescents, and glomerular sclerosis. Sections were also assessed for the presence of tubulopathy, interstitial fibrosis, tubular atrophy, and tubulointerstitial cell infiltrates. For glomeruli pathology scoring a semi-quantitative score was used to evaluate the degree of glomerular damage. A minimum of 50 glomeruli in each group was examined and the severity of the lesion was graded from 0 to + 3: a + 1 lesion represented an involvement of ≤ 30% of the glomerulus, + 2 of 30–60%, while a + 3 lesion indicated ≥ 60% of the glomerulus was involved. Measurement of GBM thickness was performed on glomerular electron micrographs of WT and *Clr*-*f*^−/−^ using Image J V1.53a^47^. PAS-stained renal tissue from two *Rag1*^−/−^ and two *Rag1*^−/−^*Clr*-*f*^−/−^ mice was additionally scored blindly for the presence of the pathological features above, and the quantified features were calculated from the mean four kidney sections per mouse.

### Electron microscopy

Processing tissue for transmission electron microscopy followed the published protocol^48^. Briefly, small pieces of kidney tissues from 12-week-old WT and *Clr*-*f*^−/−^ mice were fixed in 2.5% glutaraldehyde solution and then processed using the 1% osmium tetroxide/uranyl acetate method. Samples were embedded in Epon Araldite resin before cutting using a Reichert-Jung ultracut E ultramicrotome. Samples were visualized on a JEOL JEM 1230 transmission electron microscope and images captured using a Hamamatsu ORCA-HR digital camera.

### Urine and plasma chemical waste and electrolyte measurement

Approximately 150 μL of urine was collected daily from 12-week-old male WT and *Clr*-*f*^−/−^ mice at the same time of the day for 5 consecutive days. Urine was centrifuged at 10,000 rpm to remove debris and then stored at − 80 °C. Facial vein blood was collected from the same mice in heparinized tubes and isolated by centrifugation at 3000*g* for 5 min and stored at − 80 °C. Urine and plasma samples were sent to Idexx Laboratories (Markham, Ontario) for protein, creatinine, and electrolyte concentration measurements.

### Blood pressure measurements on 12 and 24-week-old mice

Systolic and diastolic blood pressures (BPs) were measured by tail-cuff plethysmography on 12 and 24 week-old male WT and *Clr*-*f*^−/−^ mice at steady state using a BP2000 Visitech model. BP was measured daily in the same room and at the same hour of the day for 5 consecutive days. BP values of the last 3 consecutive measurements were used to calculate the mean BP value as the first 2 days were considered essential for mice adaptation.

### Kidney RNA sequencing

For kidney RNA-seq, total RNA was isolated from freshly frozen kidneys of three age- and sex-matched WT and *Clr*-*f*^−/−^ mice at 7, 13, and 24 weeks of age using TRIzol (Thermo Fisher Scientific) and RNA Clean & Concentrator-5 kit (Zymo Research, Irvine, CA, USA). RNA samples were sent to Genome Quebec Innovation Centre, McGill University, Montreal, QC for library preparation and RNA-seq. RNA-seq was performed on the Illumina HiSeq 4000 platform, with paired-end reads, at > 30 × 10^6^ read depth per sample.

### RNA-seq analysis

The quality of the sequencing reads was inspected using FastQC tool V0.11.5 (http://www.bioinformatics.babraham.ac.uk/projects/fastqc/) and reads with Phred quality score of 30 over 90% of the reads were retained utilizing the Fastx-toolkit V0.0.13 (http://hannonlab.cshl.edu/). Next, the high-quality reads were mapped to the mouse GENCODE M25 annotation database (GRCm38.p6)^49^ using the STAR aligner V2.7.5a^50^. STAR aligner was then used to remove duplicates, create transcriptome hits count tables, and generate wiggle files. Subsequently, DESeq2 package V1.28.1^51^ was used to remove hits with CPM values less than 1 and calculate differentially expressed genes with FDR-transformed *P*-value > 0.05 and log fold change cutoff of 1.5. FPKM values for all time points and samples are provided (Supplementary Dataset File 1).

### Functional enrichment analysis

Gene ontology and Functional Enrichment Analysis was performed using g:Profiler (version e102_eg49_p15_7a9b4d6) with g:SCS multiple testing correction method applying significance threshold of 0.05 (https://biit.cs.ut.ee/gprofiler/gost)^52^ and the PANTHER Classification System with Fisher’s Exact testing and FDR threshold of < 0.05 (http://pantherdb.org/citePanther.jsp)^53^. Functional enrichment networks were created using gene sets of GO Biological Processes (GO:BP) for Mus Musculus (GRCm38.p6) BioMart release: 2020-12-08 downloaded from g:Profiler (https://biit.cs.ut.ee/gprofiler/gost). Gene Set Enrichment Analysis (GSEA) was performed using GSEA V4.03^54,55^ using Network enrichment based on FDR < 0.05. Networks were assembled, curated, and visualized using Cytoscape V3.7.1^56^. Heatmaps were generated and clustered by Euclidian distance using Morpheus (https://software.broadinstitute.org/morpheus).

### Abdominal and ectopic lipid analysis

Fat accumulation was determined by the weight of abdominal fat dissected from individual mice. Bodyweight measurements were taken before fat extractions. For Oil Red O (ORO) staining, frozen kidney sections were cut at 10 μm using a cryostat then air-dried for 30 min. ORO staining was performed by fixing the sections with 3 washes of 60% isopropanol, the incubating them with a working concentration (66%) of ORO solution (Sigma-Aldrich, St. Louis, MO, USA) staining for 15 min. Slides were then washed quickly with 60% isopropanol, quickly stained with Mayer’s hematoxylin to highlight nuclei, and then mounted using VectaMount AQ Aqueous Mounting Media (Vector Laboratories).

### Pathotype analysis

Pathotype analysis was performed using Differentially Expressed Genes (DEGs) with q < 0.05 identified from RNA-seq of WT and *Clr*-*f*^−/−^ 13-week old mouse kidneys. Significant *Clr*-*f*^−/−^ DEGs with identifiable ENSEMBL human orthologs were compared with DEGs of 15 human kidney disease expression sets (disease versus healthy kidney)^57–64^ downloaded from Nephroseq database at (http://www.nephroseq.org) (Supplementary Dataset File 2)^65^. *Clr*-*f*^−/−^ mouse and human kidney disease DEG sets were hierarchically clustering by Euclidean distance and graphed in a similarity matrix using the Morpheus software (https://software.broadinstitute.org/morpheus). Functional enrichment using g:Profiler, (using parameters described above) was performed on four groups of expression clusters with corresponding increased and decreased DEGs and non-corresponding DEGs from the compared expression profiles of *Clr*-*f*^−/−^ and IgA nephropathy (IgAN) kidney disease.

### Flow cytometry

Kidneys from age- and sex-matched WT and *Clr*-*f*^−/−^ mice were harvested, homogenized in a Petri dish on ice with RPMI media containing collagenase type 4 (2 mg/mL) (Fisher), and incubated at 37 °C for 30 min with gentle agitation. Digested tissue was passed through a 70-μm nylon filter, PBS washed, and the remaining red cells were lysed with ACK lysing buffer for 5 min on ice. Kidney immune cells were isolated by RT centrifugation at 2000 rpm for 30 min in 40% Percoll. The cell pellet was resuspended in PBS and cells counts were obtained by hemocytometer. Various mAbs were used to verify cell-surface protein expression, cells were stained for flow cytometry analysis with fluorochrome-labelled mouse antibodies: anti-CD45 (eBioscience, clone 30-F11), anti-TCRβ (eBioscience, clone H57-597), anti-NK1.1 (eBioscience, clone PK136), anti-CD19 (BioLegend, clone 1D3/CD19), anti-CD11b (BioLegend, clone M1/70), anti-F4/80 (BioLegend, clone BM8), anti-MHCII(I-A/I-E) (BioLegend, clone M5/114.15.2), anti-Ly6G (BioLegend, clone 1A8), anti-NKp46 (eBioscience, clone 29A1.4), and isotype controls. Cell viability was determined using a Fixable Viability Dye (eBioscience, Cat# 65-0865-14). To identify and quantify immune cell populations in the WT and *Clr*-*f*^−/−^ kidneys, multiparametric flow cytometry analyses using antibodies against cell-specific markers were used to identify neutrophils (CD11b^+^Ly6G^+^), macrophages (CD11b^+^Ly6G^−^F4/80^+^MHCII^+^), NK cells (CD11b^lo^Ly6G^−^TCRβ^−^NK1.1^+^), and NKp46^+^ cells (CD11b^lo^Ly6G^−^TCRβ^−^NKp46^+^), T cells (Ly6G^−^NK1.1^−^TCRβ^+^) and B cells (CD11b^−^Ly6G^−^TCRβ^−^CD19^+^). All samples were acquired on a BD-Fortessa flow cytometer.

### Statistics

Statistical significance was determined by Student *t* test where applicable, with *P* values reported. GBM measurements utilized a one-way ANOVA analysis with Sidak’s correction for multiple comparisons, respectively.

### Ethical standards

All animal investigations in this study were conducted in compliance with the standards of Dalhousie University and the University of Ottawa animal ethics care committees and the Canadian Animal Care Council.

## Supplementary Information

Supplementary Figures.

Dataset S1.

Dataset S2.

## Data Availability

RNA sequencing data will be deposited in Gene Expression Omnibus (GEO) at accession number: GSE198814 prior to publication.
