# Supplementary material for: Clr-f expression regulates kidney immune and metabolic homeostasis
Source: Sci Rep. 2022 Mar 22;12:4834. doi: 10.1038/s41598-022-08547-9 (PMC8940912; doi:10.1038/s41598-022-08547-9)
Supplement: Supplementary file 1 — Supplementary Figures. [file 41598_2022_8547_MOESM1_ESM.pdf]

SUPPLEMENTARY MATERIAL
Scientific Reports

Clr-f Expression Regulates Kidney Immune and Metabolic Homeostasis.

Haggag S. Zein, Elias Abou-Samra, Michal Scur, Alex Gutsol, Clayton W. Hall, Bishal Dasgupta, Lara Gharibeh, Turki Abujamel, Daniel Medina-Luna, Gayani S. Gamage, Tessa J. Pelino, Mona Nemer, Mir Munir A. Rahim, Alexander Steinle, Brendon D. Parsons, and Andrew P. Makrigiannis

Correspondence: andrew.makrigiannis@dal.ca

Fig.S1 Clr-f mutagenesis validation
Fig.S2 Clr-f and NKR-P1G expression analysis
Fig.S3 Full-length gel images

SUPPLEMENTARY FIGURES

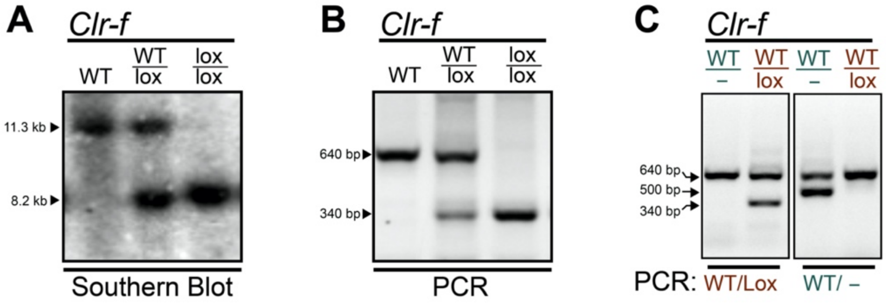

Fig. S1

Fig. S1 Clr-f mutagenesis validation. (**A**) Southern blot analysis of founder mice derived from targeted ES cells to identify *Clr-f*^{lox} mice. (**B)** PCR analysis validation of *Clr-f*^{lox} mouse generation using WT, Neo, and Reverse PCR primers indicated in panel A. (**C**) PCR analysis of tail DNA from *Clr-f*$^{wt/lox}$ and *Clr-f*$^{wt/-}$ mice generated from a cross with CMV-Cre transgenic mice. Cropped gel images of separate gels are shown. Full-length gel images are shown in Fig. S3B-D.

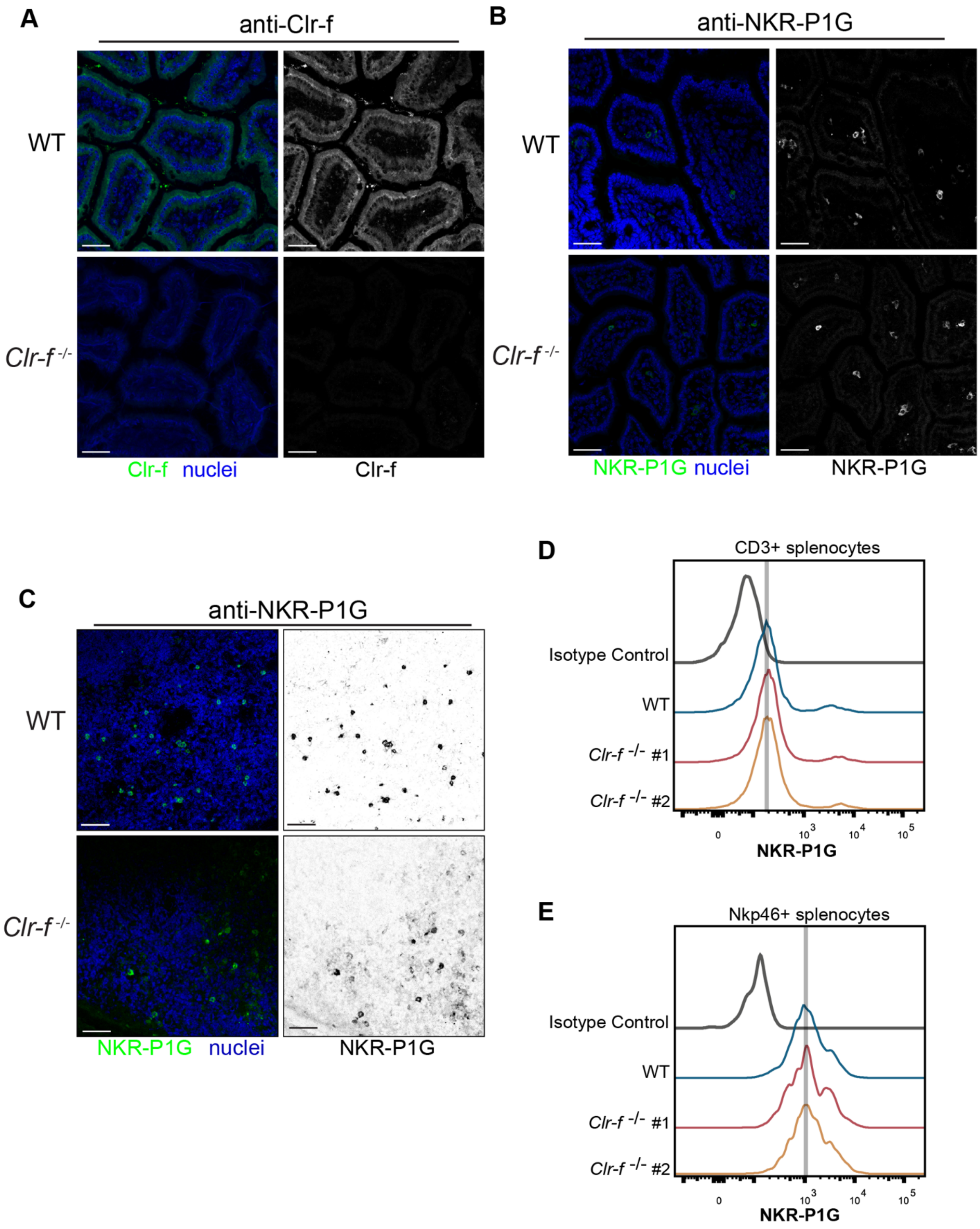
A
anti-Clr-f
WT
Clr-f -/-
Clr-f nuclei
Clr-f
B
anti-NKR-P1G
WT
Clr-f -/-
NKR-P1G nuclei
NKR-P1G
C
anti-NKR-P1G
WT
Clr-f -/-
NKR-P1G nuclei
NKR-P1G
D
CD3+ splenocytes
Isotype Control
WT
Clr-f -/- #1
Clr-f -/- #2
0
10 3
10 4
10 5
NKR-P1G
E
Nkp46+ splenocytes
Isotype Control
WT
Clr-f -/- #1
Clr-f -/- #2
0
10 3
10 4
10 5
NKR-P1G

Fig. S2

Fig.S2 Clr-f and NKR-P1G expression analysis. (**A**) IF images of small intestine tissues of WT and *Clr-f*$^{-/-}$ mice using an anti-Clr-f antibody (10A6) and (**B**) anti-NKR-P1G antibody (8A10). (**C**) IF images of spleen tissues of WT and *Clr-f*$^{-/-}$ mice using anti-NKR-P1G antibody. Scale bars represent 40μm. Flow cytometry analysis of NKR-P1G expression on (**D**) CD3+ splenocytes and (**E**) NKp46+ splenocytes from WT and *Clr-f*$^{-/-}$ mice.

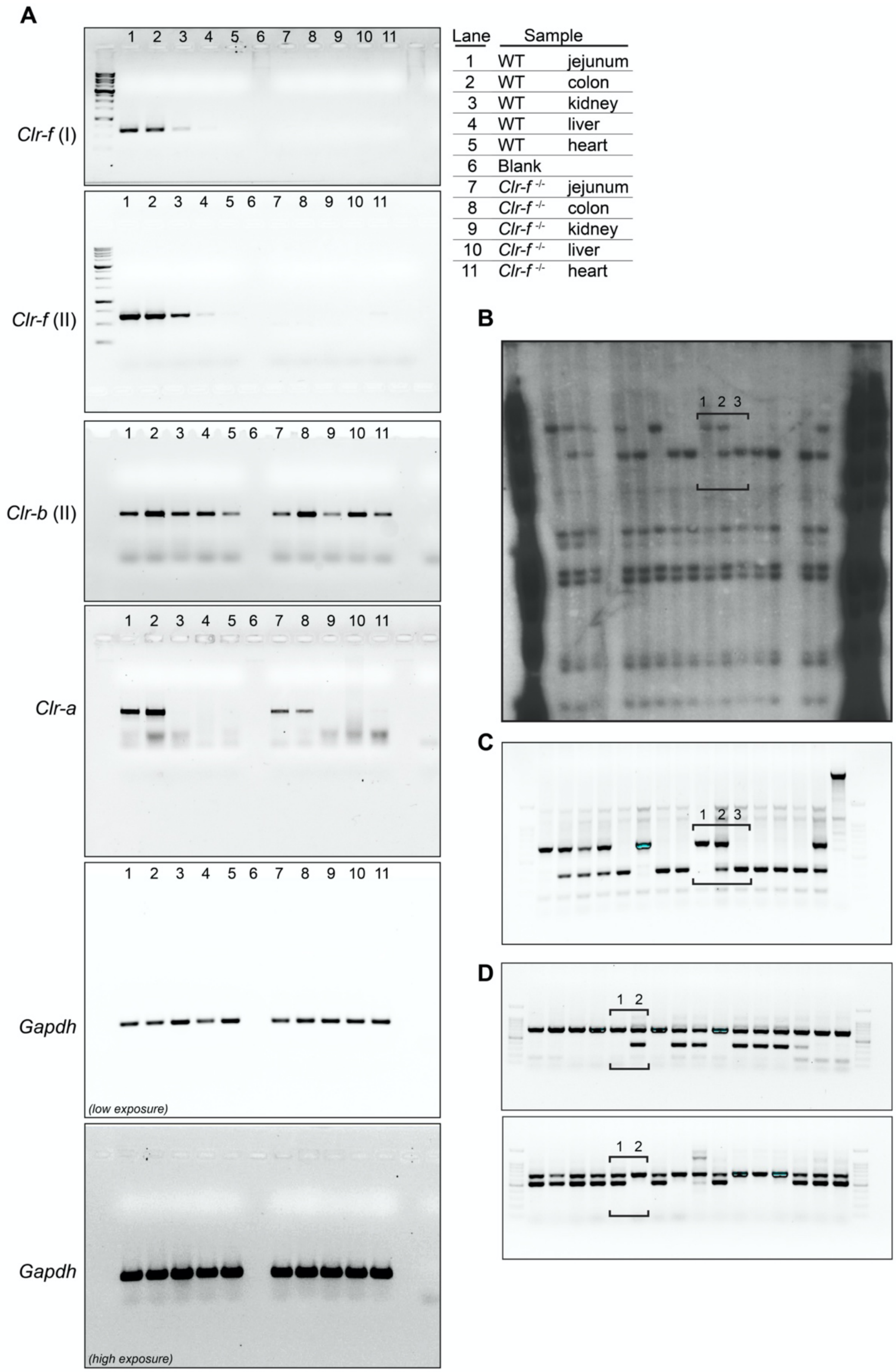

A

Lane	Sample	
1	WT	jejunum
2	WT	colon
3	WT	kidney
4	WT	liver
5	WT	heart
6	Blank	
7	*Clr-f* $^{-/-}$	jejunum
8	*Clr-f* $^{-/-}$	colon
9	*Clr-f* $^{-/-}$	kidney
10	*Clr-f* $^{-/-}$	liver
11	*Clr-f* $^{-/-}$	heart

B

Lane	Sample
1	*Clr-f* ^{WT}
2	*Clr-f* $^{WT/lox}$
3	*Clr-f* $^{lox/lox}$

C

Lane	Sample
1	*Clr-f* ^{WT}
2	*Clr-f* $^{WT/lox}$
3	*Clr-f* $^{lox/lox}$

D

Lane	Sample
1	*Clr-f* $^{WT/-}$
2	*Clr-f* $^{WT/lox}$

Fig. S3

Fig.S3 Full-length Gels (**A**) Full-length gel images of RT-PCR analysis shown in Fig.1C (**B**) Full-length gel image of Southern blot analysis data shown in Fig.S1A. (**C** and **D**) Full-length gel image of PCR analysis validation of *Clr-f* $^{wt/lox}$ and *Clr-f* $^{wt/-}$ mouse generation shown in Fig.S1B and Fig.S1C, respectively.
